# 

**Published:** 1882-09

**Authors:** 


					﻿vol. II.
SEPTEMBER, 1882,
NO. 9.
THE
HOMEOPATHIC PHYSICIAN,
A MONTHLY JOURNAL OF MEDICAL SCIENCE.
“Jfagrna est ver Has, el prcevalebit."
ZHL CT. LIE IE, ZMZ. JD.
’ EDITOR.
CONTENTS:
(See inside of Cover.)
PHILADELPHIA:
No. 2109 CHESTNUT STREET.
r> O 2sr x> O IT.
ALFRED HEATH & CO., Sole Agents for Great Britain,
114 Ebury Street, Eaton Square.
Yearly Subscription, $2.00, invariably in advance;
Entered at the Philadelphia Post-Office, as Second Class Mail Matter.
				

